# Soil Biological Responses under Different Vegetation Types in Mediterranean Area

**DOI:** 10.3390/ijerph19020903

**Published:** 2022-01-14

**Authors:** Speranza Claudia Panico, Valeria Memoli, Lucia Santorufo, Stefania Aiello, Rossella Barile, Anna De Marco, Giulia Maisto

**Affiliations:** 1 Dipartimento di Biologia, Università Degli Studi di Napoli Federico II, Via Cinthia, 80126 Napoli, Italy; speranzaclaudia.panico@unina.it (S.C.P.); lucia.santorufo@unina.it (L.S.); aiello.stefania@hotmail.it (S.A.); giulia.maisto@unina.it (G.M.); 2 BAT Center—Interuniversity Center for Studies on Bioinspired Agro-Environmental Technology, University of Naples Federico II, 80126 Naples, Italy; ademarco@unina.it; 3 Parco Nazionale del Vesuvio, Via Palazzo del Principe c/o Castello Mediceo, 80044 Ottaviano, Italy; rbarile@epnv.it; 4 Dipartimento di Farmacia, Università degli Studi di Napoli Federico II, Via Montesano, 80131 Napoli, Italy

**Keywords:** integrative biological responses index, unburnt and burnt soils, *Pinus pinea* L., *Quercus ilex* L., *Robinia pseudoacacia* L., herbaceous species

## Abstract

The knowledge of the effects of fire on soil properties is of particular concern in Mediterranean areas, where the effects of vegetation type are still scarce also. This research aimed: to assess the properties of burnt soils under different vegetation types; to highlight the soil abiotic properties driving the soil microbial biomass and activity under each vegetation type; to compare the biological response in unburnt and burnt soils under the same vegetation type, and between unburnt and burnt soils under different vegetation types. The soils were collected at a Mediterranean area where a large wildfire caused a 50% loss of the previous vegetation types (holm oak: HO, pine: P, black locust: BL, and herbs: H), and were characterized by abiotic (pH, water, and organic matter contents; N concentrations; and C/N ratios) and biotic (microbial and fungal biomasses, microbial respiration, soil metabolic quotient, and hydrolase and dehydrogenase activities) properties. The biological response was evaluated by the Integrative Biological Responses (IBR) index. Before the fire, organic matter and N contents were significantly higher in P than H soils. After the fire, significant increases of pH, organic matter, C/N ratio, microbial biomass and respiration, and hydrolase and dehydrogenase activities were observed in all the soils, especially under HO. In conclusion, the post-fire soil conditions were less favorable for microorganisms, as the IBR index decreased when compared to the pre-fire conditions.

## 1. Introduction

The above-ground and below-ground interactions play a fundamental role in controlling terrestrial ecosystem processes and properties. Though plants provide the food resources for the decomposer and for the root-associated organisms, decomposers regulate nutrient availabilities by dead plant material degradation [1]. However, a fundamental role in regulating nutrient supply is also played by the soil chemical, biochemical, and physico-chemical properties [2]. Due to the intimate interactions, specific relationships occur among dominant plant species and soil microbial components in terrestrial ecosystems [3,4]. In fact, a great part of the variation in soil microbial communities is explained by plant community composition and soil chemistry [5], but little is known about the links between below-ground and above-ground components [6].

Mediterranean ecosystems are characterized by a wide heterogeneity of vegetation ranging from pine and holm oak (*Quercus ilex* L.) forests to shrublands and grasslands, which differently influence soil microbial community. For example, herbaceous species, producing labile and easily decomposable litter, have less potential carbon storage than trees [7], favoring a bacterial-based soil food web [8], whereas the complex litter of trees supply several carbon sources, hosting a diversified microbial community that influences the organic matter decomposition rate and turnover [9]. In addition, in Mediterranean environments, several invasive species, such as black locust trees (*Robinia pseudoacacia* L.), are becoming widespread, with important alterations on the native soil microorganism community composition [10]. Despite the Mediterranean forests being subjected to specific constraints, such as drought stress, heat waves, low nutrient availability, and recalcitrant organic compounds, there is still a lack in the understanding of factors controlling microbial abundance and activities [11].

In Mediterranean areas, fire events are recognized as fundamental ecological factors to maintain the biodiversity of the ecosystem; however, these events are becoming more and more frequent, and can sometimes cause a great loss of biodiversity, and can compromise the soil functionality. Fires, in fact, which modify the plant community composition, influence the quantity and quality of food resources added to the soil, with consequences on the community and activities of microorganisms [12].

In the last decades, specific indicators of soil microbial activity, such as enzymatic activities (hydrolase and dehydrogenase), soil respiration, and metabolic quotient, have been proposed [13,14,15]. More recently, many authors state that the use of a single property to assess fire changes on soil biological properties is not enough [16,17]. The use of integrated indices is becoming more common, as they provide overall interpretations of the biological responses to the abiotic environmental factors [18,19,20]. Particularly, the Integrative Biological Responses (IBR) index, proposed by Beliaeff and Burgeot [21], is often used to evaluate the biological responses resulting from in situ perturbation on environmental matrices [22,23].

Therefore, the aims of the research were: (i) to assess the abiotic and biotic properties of soils under different vegetation types after the fire; (ii) to highlight the soil abiotic properties driving the soil microbial biomass and activity under each vegetation type; (iii) to compare the biological response in unburnt and burnt soils under the same vegetation type; (iv) to evaluate differences in the biological response in unburnt and burnt soils under different vegetation types.

To achieve the aims, the soils were characterized by abiotic (pH, water and organic matter contents, N concentrations, and C/N ratios) and biotic properties (microbial and fungal biomasses, microbial respiration, soil metabolic quotient, and hydrolase and dehydrogenase activities). In addition, the soil microbial biomass and activities were used to calculate the IBR index as a tool to evaluate the soil biological responses.

## 2. Materials and Methods

The study was carried out inside the Vesuvius National Park, Naples (Italy), established in 1995. The soils are classified as Lepti-vitric Andosols [24]. The flora of the park is typically Mediterranean, composed by patches of herbaceous species (mosses, lichens, Centranthus ruber L., Helichrysum italicum R., *Artemisia campestris* L., *Rumex scutatus* L., and many species of grasses), shrubs (such as *Myrtus communis* L., *Laurus nobilis* L., *Viburnum tinus* L., *Cistus* sp., *Ginesta* sp.), and forest areas where holm oaks (*Quercus ilex* L.) and pine species (*Pinus nigra* L., *Pinus pinea* A., *Pinus sylvestris* L., *Pinus pinaster* A.) are dominant [25,26]. Additionally, black locusts (*Robinia pseudoacacia* L.), an invasive species used since 1912 for afforestation and the stabilization of volcanic substrates [27], are widespread in many areas of the Vesuvius National Park.

In June 2017, the Vesuvius National Park was affected by an undesired human fire [10]. The burnt area was affected by a severe surface fire (level 4 on the Soil Burn Severity index) that caused the loss of more than 50% (approximately 3000 ha) of the existing plant cover, and a complete consumption of the forest floor [28,29].

The soil samples were collected in two sampling campaigns: one year before (2016) and one year after (2018) the wildfire occurred in 2017, and are named, respectively, BF and AF. Surface (0–10 cm) BF soils were randomly collected at ten sites: five covered by herbaceous vegetation (H_1-5), and five covered by pines (P_1-5). Surface (0–10 cm) AF soils were collected at twenty sites: ten sites were the same collected under herbaceous and pine species, BF, and ten sites were collected under different vegetation types: five covered by black locust (BL_1-5), and five were covered by holm oak (HO_1-5). The coordinates of the sampling sites and the main plant cover are reported in Table 1.

At each site, five subsamples of surface soils (0–10 cm) soils were collected after litter or ash removal, respectively, for BF and AF soils, in proximity of the plant roots, and mixed together in order to obtain a representative composite sample to perform, after sieving (<2 mm) in the laboratory, the analyses.

### 2.1. Physico-Chemical Analyses

The physico-chemical analyses were performed, in triplicates, on fresh soil samples (pH and water content (WC)), and on dried (at 105 °C, until constant weight) and pulverized (Fritsch Analysette Spartan 3 Pulverisette 0) soil samples (total C and N, and organic matter contents (OM)). Soil pH was measured according to USDA-NRCS [30] on aqueous extracts obtained by adding distilled water to soils (2.5:1 = w:w); WC was determined by gravimetrically drying fresh soil at 105 °C until a constant weight; C and N contents were analyzed by gas chromatography (Thermo Finnigan, CNS Analyzer), and used to calculate the C/N ratio; OM content was obtained by multiplying the C_org_ (measured as described for C content after soil treatment with HCl (10%) to exclude carbonates) by 1.724 [31].

### 2.2. Biological Analyses

The performed biological analyses, in triplicates, on soil samples stored at 4 °C within three days from the sampling were: the microbial carbon (C_mic_), the fungal carbon (C_fung_), basal respiration (BR), and three enzymatic activities (hydrolase (HA) and dehydrogenase (DHA)). In addition, the metabolic quotient (qCO_2_) was calculated. C_mic_ was evaluated according to Anderson and Domsch [32], whereas C_fung_ was estimated based on the determination of total fungal biomass (TFB), as described by Memoli et al. [33]. Briefly, TFB was assayed, after staining with aniline blue, by the membrane filter technique [34], determining hypha length by the intersection method [35] with an optical microscope (Optika, B-252). To obtain the fungal fraction of microbial carbon, the values of fungal biomass were converted to fungal carbon (C_fung_) on the basis of mean values reported for the C/N ratio [36] and N content [37] in fungi. BR was estimated as CO_2_ evolution from the samples at 55% of water holding capacity after incubation in tight containers for 10 days at 25 °C by NaOH absorption followed by two-phase titration with HCl [38]. The qCO_2_, the degree of activity of the microbial biomass, was calculated using the C-CO_2_ obtained by basal respiration data and microbial C [39].

HA and DHA activities were evaluated using fluorescein diacetate (1 mg mL^−1^) and 2,3,5 triphenyltetrazolium chloride 1.5%, respectively, as substrates according to Memoli et al. [40]. The results of HA and DHA were, respectively, expressed as mmol of fluorescein (FDA) and triphenyl formazan (TPF) produced in 1 min for 1 *g* of dried soil.

### 2.3. Integrative Biological Response Index (IBR)

Soil microbial characteristics, described in Section 2.3, were combined into the IBR according to Beliaeff and Burgeot [21]. In more detail, for each property, the general mean (*m*) and the standard deviation (*s*) were calculated in order to obtain Y:

Y = (X − *m*)/*s* (1)

where X was the mean value of a single property.

Then, Z was calculated as:

Z = −Y (2a)

or

Z = Y (2b)

where Equation (2a) was used in case of inhibiting effects, and Equation (2b) was used in case of stimulating effects.

In particular, C_mic_, C_fung_, BR, HA, and DHA were considered to decrease within adverse conditions, whereas qCO_2_ was assumed to increase. The score (S) was calculated as:

S = Z + |Min|(3)

where S ≥ 0, and |Min| is the absolute value for the minimum value for all calculated Y at each considered property.

Then, star plots were created to show the score results (S), and to calculate the

IBR as:

(4) $$\mathrm{IBR}=\sum_{i=1}^{n}Ai$$

where *A_i_* =$\frac{S_{i}}{2\;}\sin\beta \;(S_{i}\cos\beta +S_{i+1\;}\sin\beta )\;\mathrm{and}\;\beta =\arctan\left(\frac{S_{i+1\;}\sin\alpha }{S_{i}-S_{i+1\;}\cos\alpha }\right)$, and corresponded to the area connecting two scores; *S_i_* and *S_i+_*_1_ were two consecutive clockwise scores (radius coordinates) of a given star plot; α = 2π/*n*, where *n* is the number of investigated biological properties.

The positioning of the properties, based on their similarity around the star plot, influences the IBR values [21].

### 2.4. Statistical Analyses

The normality of the distribution of the data sets was assessed by the Shapiro–Wilk test.

The paired *t*-tests or the signed rank tests, according to the normal or non-normal distribution of the datasets, were performed to evaluate the significance of the differences between soils collected before fire (BF), and one year after fire (AF).

Pearson’s or Spearman’s tests, according to the normal or non-normal distribution of the datasets, were performed to evaluate the relationships between soil abiotic and biotic properties in both BF and AF soils.

The ANOVA test or the signed rank tests, according to the normal or non-normal distribution of the datasets, were performed to compare the significance of the differences among different plant covers in AF soils.

The performed statistical tests were considered significant when *p* < 0.05.

The principal component analysis (PCA) was performed using all the investigated properties of AF soils in order to test the site distribution in the principal component (PC) space.

The graphs were created by SigmaPlot12 software (Jandel Scientific, San Rafael, CA, USA). The univariate statistical tests were performed using the Systat_SigmaPlot_12.2 software (Jandel Scientific, San Rafael, CA, USA), whereas the PCA was performed using the Vegan^ package (R Core Team, 2016).

## 3. Results

### 3.1. Soil Physico-Chemical and Biological Properties before the Fire

**Figure 1:**
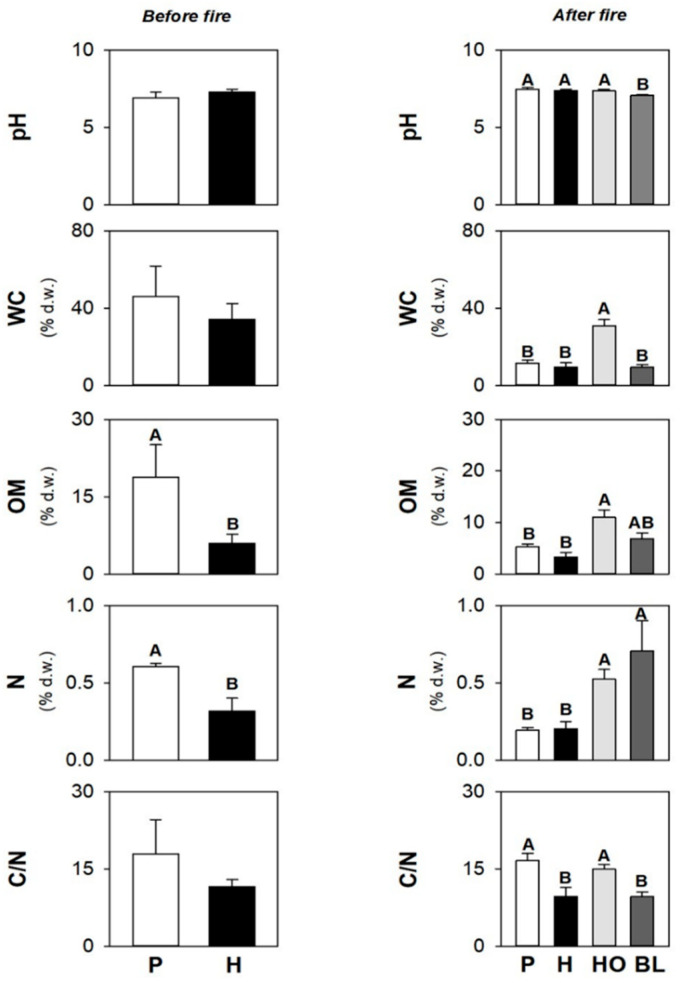
Mean values (±s.e.) of pH, water content (WC), organic matter content (OM), N concentration, and C/N ratio of soil sampled under pine (P) and herbs (H) before the fire and under the pine (P), herbs (H), holm oak (HO), and black locust (BL) after the fire. Different uppercase letters indicate statistically significant differences (*p* < 0.05) among different vegetation types in soils collected before and after the fire.

Before the fire, pH was weakly alkaline (Figure 1) in both soils collected under pines (P) and herbaceous species (H); water content (WC) was, on average, 46.2% d.w. in P soils, and 34.2% d.w. in H soils (Figure 1); organic matter (OM) content and N concentrations were statistically higher in P (OM: 18.7% d.w. and N: 0.61% d.w) than in H (OM: 11.5% d.w. and N: 0.01% d.w.) soils (Figure 1); C/N ratios were 17.8 and 11.5, respectively, in P and H soils (Figure 1).

**Figure 2:**
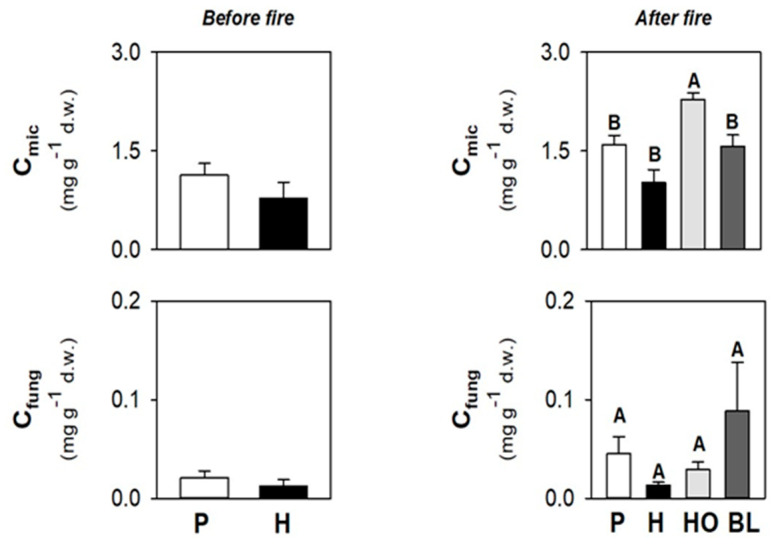
Mean values (±s.e.) of C_mic_ and C_fung_ of soil sampled under pine (P) and herbs (H) before the fire and under the pine (P), herbs (H), holm oak (HO), and black locust (BL) after the fire. Different uppercase letters indicate statistically significant differences (*p* < 0.05) among different vegetation types in soils collected before and after the fire.

The microbial carbon (C_mic_) was, on average, 1.1 and 0.8 mg g^−1^ d.w., respectively, in P and H soils (Figure 2); the fungal carbon (C_fung_) was 0.1 and 0.2 mg g^−1^ d.w., respectively, in P and H soils (Figure 2).

**Figure 3:**
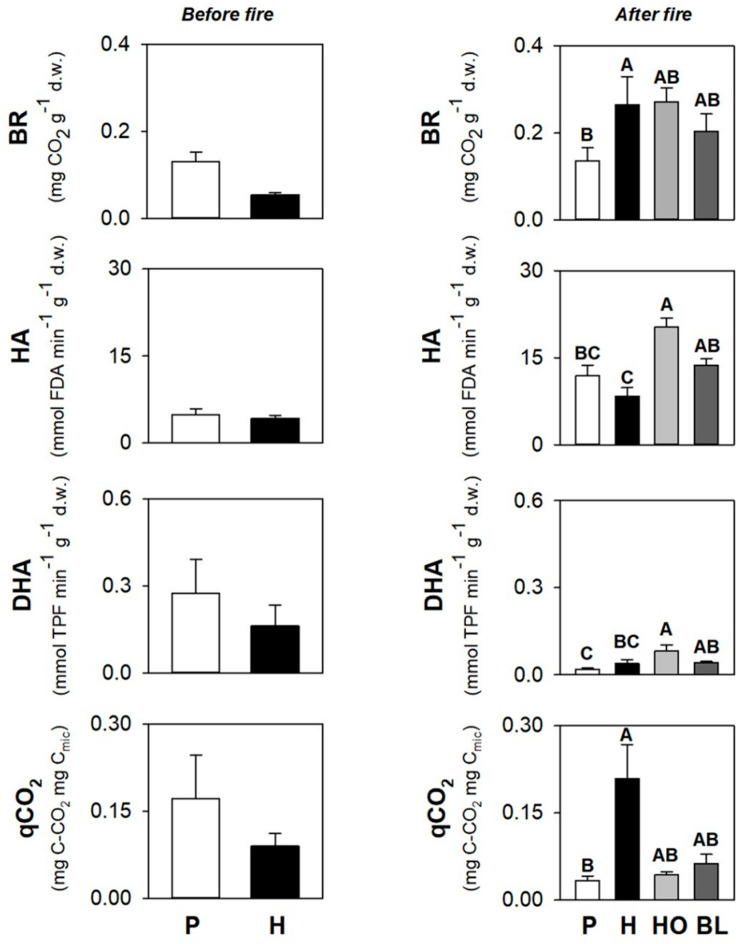
Mean values (±s.e.) of basal respiration (BR), hydrolase (HA) and dehydrogenase (DHA) activities, and metabolic quotient (qCO_2_) of soil sampled under pine (P) and herbs (H) before the fire and under the pine (P), herbs (H), holm oak (HO), and black locust (BL) after the fire. Different uppercase letters indicate statistically significant differences (*p* < 0.05) among different vegetation types in soils collected before and after the fire.

The basal respiration (BR) was 0.13 and 0.05 mg CO_2_ g^−1^ d.w., respectively, in P and H soils, with statistically significant differences (Figure 3); the hydrolase (HA) activity was 4.94 and 4.28 mmol FDA min^−1^ g^−1^ d.w, respectively, in P and H soils (Figure 3); the dehydrogenase (DHA) activity was 0.27 and 0.16 mmol TPF min^−1^ g^−1^ d.w., respectively, in P and H soils (Figure 3); qCO_2_ was 0.17 and 0.09 mg C-CO_2_ mg^−1^ C_mic_, respectively, in P and H soils (Figure 3).

### 3.2. Correlations between Biotic and Abiotic Properties in P and H Soils Collected before the Fire

Before the fire, in P soils, C_fung_ was positively correlated to soil pH, whereas C_mic_ and BR were negatively correlated to soil pH (Table 2); C_fung_ and BR were negatively correlated to soil WC and OM, whereas C_mic_, qCO_2_, HA, and DHA were positively correlated to soil WC and OM (Table 2); C_fung_ and BR were positively correlated to soil N concentrations and C/N ratio, whereas C_mic_, HA, and DHA were negatively correlated to soil N concentrations and C/N ratio (Table 2); qCO_2_ was negatively correlated to soil N concentrations (Table 2).

In H soils, BR, HA, and DHA were negatively correlated to soil pH, whereas C_mic_ was positively correlated to soil pH (Table 2); C_mic_, HA, DHA, and qCO_2_ were positively correlated to soil WC, whereas C_fung_ and BR were negatively correlated to soil WC (Table 2); C_fung_ and DHA were positively correlated to soil OM, whereas C_mic_, HA, and qCO_2_ were negatively correlated to soil OM (Table 2); C_fung_, HA, DHA, and qCO_2_ were negatively correlated to soil N concentrations (Table 2); C_fung_, BR, and qCO_2_ were positively correlated to soil C/N, whereas C_mic_, HA, and DHA were negatively correlated to soil C/N (Table 2).

### 3.3. Soil Physico-Chemical and Biological Properties after the Fire

After the fire, the pH was weakly alkaline (Figure 1) in soils collected under P, H, and holm oaks (HO), and was statistically different from the neutral value detected in soils under black locusts (BL); WC was 30.9% d.w. in HO soils (Figure 1), and statistically higher than those measured in soils collected under the other vegetation types (11.7, 9.39, and 9.30% d.w., respectively, in P, BL, and H soils); OM contents and N concentrations ranged, respectively, from 3.31 to 11.0% d.w., and from 0.20 to 0.71% d.w., with statistically higher values in HO and BL soils than in P and H soils (Figure 1); C/N ranged from 9.56 to 16.6, and was statistically higher in P and HO soils (Figure 1).

C_mic_ ranged from 1.02 to 2.29 mg g^−1^ d.w., and was statistically higher in HO soils (Figure 2); C_fung_ was, on average, 0.1 mg g^−1^ d.w., and did not statistically vary under the different vegetation types (Figure 2).

BR ranged from 0.14 to 0.27 mg CO_2_ g^−1^ d.w., and statistically varied in soils under different vegetation types, with the lowest value in P soils and the highest value in H soils (Figure 3); HA activity ranged from 4.28 to 4.94 mmol FDA min^−1^ g^−1^ d.w., and statistically varied in soils under different vegetation types, with the lowest value in H soils and the highest value in HO soils (Figure 3); DHA activity ranged from 0.16 to 0.27 mmol TPF min^−1^ g^−1^ d.w., and statistically varied in soils under different vegetation types, with the lowest value in P soils and the highest value in HO soils (Figure 3); qCO_2_ ranged from 0.03 to 0.21 mg C-CO_2_ mg^−1^ C_mic_, and statistically varied in soils under different vegetation types, with the lowest value in P soils and the highest value in H soils (Figure 3).

### 3.4. PCA on Dataset after the Fire

**Figure 4:**
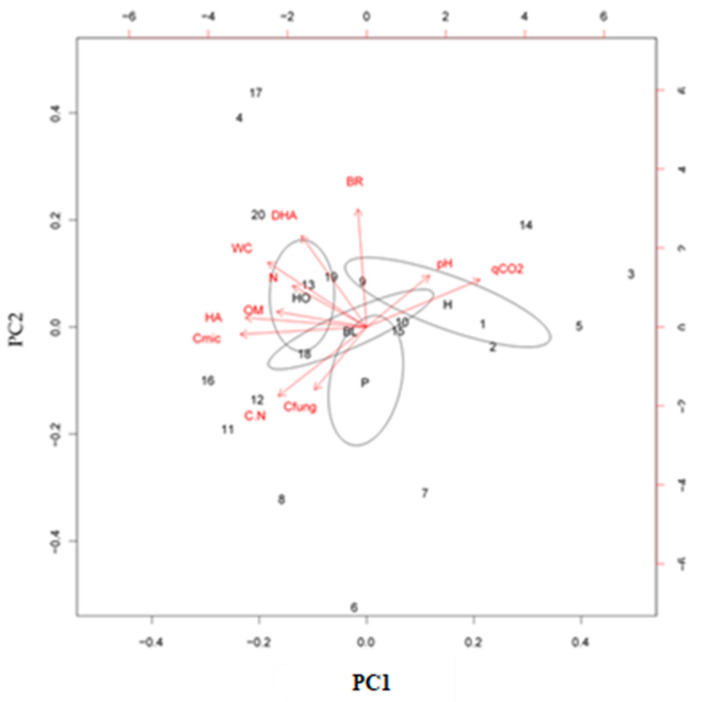
The principal component analyses (PCA) plots of abiotic (pH, WC, OM, N, and C/N) and biotic properties (BF, Cmic, BR, HA, and DHA), and their distribution in space for the soils collected after the fire (AF) under herbs (H), pine (P), black locust (BL), and holm oak (HO). Circle lines in the PCA plot are superimposed to show the sampling sites with the same vegetation type. H: soil sampled under herbs; P: soil sampled under pines species; BL: soil sampled under black locust species; and HO: soil sampled under holm oak species.

The PCA highlighted that the first two axes accounted for 58% of the total variance in the dataset (Figure 4).

The investigated soil properties were clearly separated in the PC space: pH and qCO_2_ were located in the first quadrant; C_fung_, C_mic_, and C/N were located in the third quadrant; WC, OM, N, BR, HA, and DHA were located in the fourth quadrant (Figure 4).

The site distribution in the PC space was related to plant type; particularly, along the first axis, soils covered by herbaceous (H) species were clustered in the positive direction, whereas soils covered by pines (P), black locust (BL), and holm oak (HO) were clustered in the negative direction (Figure 4). The first axis of the PCA was positively correlated to soil pH, BR, and qCO_2_, and negatively correlated to the other soil properties, whereas the second axis was positively correlated to soil pH, WC, N, C_mic_, BR, and qCO_2_, and negatively correlated to the other soil properties.

### 3.5. Correlations between Biotic and Abiotic Characteristics in P, H, OH, and BL Soils Collected after the Fire

After the fire, in P soils, all the investigated soil biotic properties, with the exception of qCO_2_, were positively correlated to soil pH (Table 3); C_fung_, BR, and DHA were negatively correlated to soil WC (Table 3); DHA was positively correlated to soil OM content (Table 3); all the investigated soil biotic properties were positively correlated to soil N concentrations (Table 3); all the investigated soil biotic properties, with the exception of HA, were negatively correlated to soil C/N (Table 2b).

In H soils, C_mic_, C_fung_, and DHA were positively correlated to soil pH (Table 3); all the investigated soil biotic properties, with the exception of DHA and qCO_2_, were positively correlated to soil WC (Table 3); all the investigated soil biotic properties, with the exception of qCO_2_, were positively correlated to soil OM content (Table 3); C_mic_, C_fung_, HA, and DHA were positively correlated to soil N concentrations, whereas qCO_2_ was negatively correlated to soil N concentrations (Table 3); all the investigated soil properties, with the exception of C_fung_ and qCO_2_, were positively correlated to soil C/N (Table 3).

In HO soils, HA was positively correlated to soil pH, whereas DHA and qCO_2_ were negatively correlated to soil pH (Table 3); C_mic_, HA, and DHA were positively correlated to soil WC, whereas C_fung_ and qCO_2_ were negatively correlated to soil WC (Table 3); BR, HA, and DHA were positively correlated to both soil OM content and N concentrations, whereas C_fung_ was negatively correlated to both soil OM content and N concentrations (Table 2b); BR and DHA were positively correlated to soil C/N, whereas C_fung_ was negatively correlated to soil C/N (Table 2b).

In BL soils, C_mic_, C_fung_, and HA were negatively correlated to soil pH, whereas BR was positively correlated to soil pH (Table 3); BR, HA, and qCO_2_ were positively correlated to soil WC (Table 2b); C_mic_, HA, and DHA were positively correlated to soil OM content, whereas C_fung_ and BR were negatively correlated to soil OM content (Table 3); all the investigated soil biotic properties were positively correlated to soil N concentration, whereas qCO_2_ was negatively correlated to soil N concentration (Table 3); C_mic_ and C_fung_ were positively correlated to soil C/N, whereas qCO_2_ was negatively correlated to soil C/N (Table 3).

### 3.6. Differences in Soil Properties under Herb and Pine Covers before and after the Fire

The comparison of the data in H or P soils before and after the fire is reported in Table 4.

In soils under H cover, the values of pH, C_mic_, BR, qCO_2_, and HA were higher in AF than BF, whereas WC, OM, N, C/N, HA, and DHA were higher in BF than AF, and C_fung_ did not show noticeable variations between BF and AF (Table 4).

In soils under P cover, the values of pH, C_fung_, C_mic_, and HA were higher in AF than BF, whereas WC, OM, N, C/N, qCO_2_, and DHA were higher in BF than AF, and BR and DHA did not show noticeable variations between BF and AF (Table 4).

### 3.7. Integrative Biological Response Index (IBR) before and after the Fire

**Figure 5:**
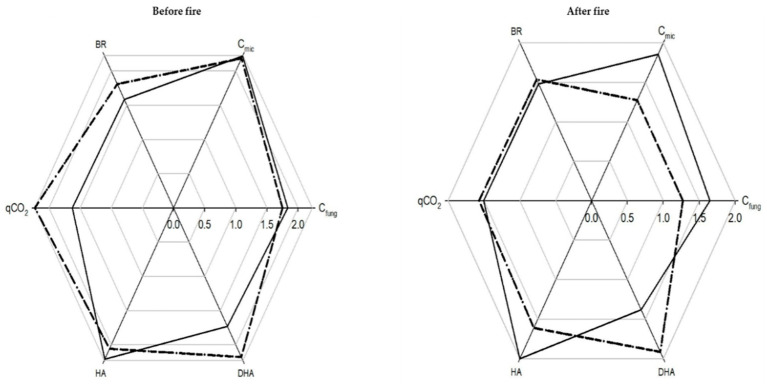
Biological response score star plot of soils collected before and after the fire under pine (dashed line) and herb (black line) species.

Before the fire, the overall IBR index was 7.70, and it was 8.44 and 7.35, respectively, for P and H soils (Figure 5).

The S scores obtained for C_mic_, C_fung_, and HA were higher for P than H soils (Figure 5); those obtained for BR, DHA, and qCO_2_ were higher for H than P soils (Figure 5).

**Figure 6:**
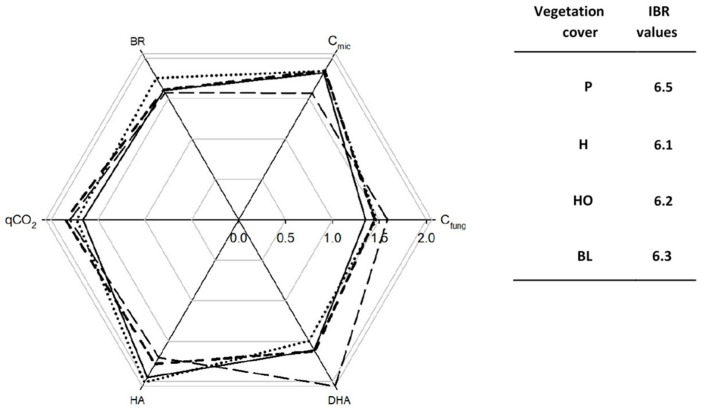
Biological response score star plot and Integrative Biological Response (IBR) of soils collected after the fire under herb (long dashed line), pine (medium dashed line), black locust (black line), and holm oak (dotted line) species.

After the fire, taking into account all the vegetation types, the overall IBR index was 6.97 (Figure 6).

The S scores obtained for C_fung_, C_mic_, and HA were higher than those obtained for BR, DHA, and qCO_2_. Instead, taking into account only P and H soils, the overall IBR index was 6.31, and it was 6.54 and 6.07, respectively, for P and H soils (Figure 6). The S scores obtained for C_fung_, C_mic_, and HA were higher for H than P soils (Figure 6); those obtained for BR, DHA, and qCO_2_ were higher for P than H soils (Figure 6).

## 4. Discussion

### 4.1. Differences in Soil Properties between Pines and Herbs before and after Fire Occurrence

Before the fire, the soil properties of the investigated Mediterranean area slightly varied between herbs (H) and pine (P) covers. In fact, only organic matter content and N concentrations were significantly higher in P than H soils. These differences could be due to the major amount of plant debris accumulated under P compared to H soils, which likely was responsible for the highest soil nutrient concentrations [41]. The abundance of litter in P soil may be explained by the slow degradation of pine needles, as commonly reported [42,43]. This hypothesis is also corroborated by Shedayi et al. [44], who found that litter accumulated under pines had greater concentrations of carbon and nitrogen as compared to those of herbaceous species. In the studied area, the greatest organic matter amount under pines could also be due to the higher plant density as compared to herbs. Besides, litter inputs, deriving from leaching phenomena along the slope [45], cannot be excluded, as pine stands are located at a lower altitude than the herbaceous ones.

The lack of significant differences between soils collected under pines and herbaceous species suggests the existence of a steady state of the plant-soil system that partially hid the effects of each vegetation type (P and H) on soil properties (i.e., WC, pH, C/N, C_mic_, C_fung_, BR, HA, DHA, qCO_2_). In fact, after a long time without any perturbations, the soil system could be characterized by a steady state with slow and undetectable changes.

However, specific relationships among soil biotic and abiotic properties were observed under pine and herbs, suggesting the soil microbial biomass and activity were affected by micro-environmental conditions, and were controlled by some abiotic properties driven by plants [7].

Moreover, in the investigated area, C_mic_ showed variations according to the soil pH measured in soils under P, as it decreased as the soil pH increased, confirming that low values of pH enhance the bacteria distribution and composition in soils of coniferous forests [46]. Before the fire, soil OM content and N concentrations also affected, in the opposite way, some biotic properties according to different vegetation types. This highlighted the fundamental roles of organic compounds and nitrogen as resources for soil microorganism growth and activity [47], which, in turn, are involved in C and N cycles [7,48,49], and in soil organic matter stabilization [7]. Instead, the other soil abiotic properties, such as WC and C/N ratios, affected, in the same direction, the soil microbial biomass and activity under both P and H covers, showing a minimization of the effects due to the two different vegetation types.

The IBR index approach highlighted that the soils under pine present better conditions for the microbial community as compared to those under herbs, as the IBR indices were, respectively, 8.44 and 7.35. The analyzed microbial biomass and HA better respond to the soil characteristics under P soils, whereas the other microbial activities respond better under H soils. The lower values of soil properties observed after the fire in P and H soils suggest that a sudden decrease of the organic matter content and, likely, of its labile fraction, occurred [50]. This was particularly true for soils under pines, agreeing with other researchers [51,52] who found that, in Mediterranean maquis, fire can cause immediate changes that can persist for several years. The microbial biomasses in burnt H and P soils slightly varied as compared to those in the unburnt ones. An exception was C_fung_ in P soils, which significantly increased after the fire, suggesting that the fire favored fungi as compared to bacteria [10]. Instead, the different behaviors of HA, extracellular enzymes, and DHA, and the intracellular enzymes between unburnt and burnt soils, suggest deep changes in different functional groups of microorganisms [53]. It can be supposed that after the fire, soil conditions are more disturbed, especially in H soils, as a conspicuous increase in BR and qCO_2_ occurred [54].

The IBR indices calculated before and after the fire highlighted that the fire event negatively affected the edaphic community responses in both H and P soils. Finally, the biological responses of P soils seemed to be more impacted by the fire (IBR = 8.44 and 6.54, respectively, before and after the fire), as the IBR index decreased more than in H soils (IBR = 7.35 and 6.07, respectively, before and after the fire). The higher S scores of qCO_2_, a stress indicator, in P soils corroborated this hypothesis. The IBR indices suggest the biological response in recovering the pre-fire conditions is faster for species typical of the early stages (herbs) of the ecological succession as compared to those of the mature ones (trees). Even though the recovery is a function of many intrinsic and extrinsic variables, a greater capability is known for species at the early stages of the ecological succession, although their mortality rates can be high [55]. Furthermore, plant recovery also depends on their vulnerability to new stressors [56].

### 4.2. Impacts of Fire and Vegetation Type on Soil Properties after Fire Occurrence

After the fire, the significant differences of both abiotic and biotic properties among soils under each vegetation type (i.e., H, P, BL, and HO) were numerous, suggesting a fundamental role of fire in modifying soil properties. However, the impact of fire on soil properties varied according to the different vegetation types, as fire and vegetation interact with each other in influencing the soil system. In fact, in the short-term after the fire (one year since the fire), plants play a fundamental role in creating new micro-habitats for the edaphic community [57,58,59]. In more details, after the fire, pH values increased as compared to before, particularly in soils under evergreen trees (P and HO) and herbs (H). The role of fire in increasing soil pH is widely reported in evergreen stands, due to the release of aliphatic compounds during litter combustion [60,61]. The highest WC observed in HO soils was probably due to the high amount of organic matter, known to increase water retention. In fact, holm oak debris has a high capability to hold water [62]. Besides, in the investigated area, the litter layer accumulated after the fire under holm oak canopies could be responsible for a low incidence of solar radiation that generated an increase of soil moisture [63]. Notwithstanding the high OM content, the higher C/N ratios in P and HO soils indicate the scarce quality of litters deriving from sclerophyllous leaves, which are rich in complex compounds, such as cellulose, hemicellulose, and lignin [64,65,66]. After the fire, the highest values of the investigated abiotic properties were often detected in HO soils, with the exception of N concentrations, which, instead, were higher in BL soils. These results could depend on the natural supplying of nitrogen, deriving from the symbiotic association between black locust roots and nitrogen-fixer bacteria [10,27,67].

After the fire, despite numerous abiotic properties significantly varying, microbial and fungal biomasses slightly changed among soils under different vegetation types. The only exception was found for HO soils, where the microbial biomass and microbial activities (i.e., BR, HA, and DHA) were significantly higher than those observed for the other soils (i.e., H, P, and BL), suggesting that the fire impacted these soils to a lesser extent than the other vegetation types. In addition, a clear separation of bacteria and fungi of ecological preferences could be supposed, as the former were enhanced (in terms of biomass and activity) by high OM, WC, and N concentrations, and the latter were enhanced by C/N ratios. The abundance of bacteria and the high rate of activities suggest their involvement in the carbon cycle in the early stages of decomposition [68]. Particularly, DHA plays an important role in the initial stages of the oxidation of soil organic matter by transferring electrons or hydrogen ions from substrates to acceptors [69,70]. Although soil microbial biomasses slightly varied among P, H, and BL soils, the microbial activities significantly varied, suggesting that different plant types cause the diversification and specialization of soil microbial communities. Thus, the plants modified some soil abiotic properties, which, in turn, were responsible for the different microbial activities [71,72], as highlighted by the numerous correlations found.

Overall, taking into account the investigated soil properties, a clear separation of H soils from the soils covered by trees (BL, P, and HO) was observed, as also shown by the site distribution into the PC space of the investigated burnt soils. According to the PCA, the main drivers of site separation were pH, WC, N, C_mic_, BR, and qCO_2_. Particularly, H soils separated from the soils covered by trees (i.e., BL, P, and HO), and were characterized by low values of WC, N, and C_mic_, and high values of pH, BR, and qCO_2_; instead, the other soils were characterized by opposite trends.

After the fire, the IBR indices showed comparable values (6.1–6.5) among H, P, BL, and OH soils, suggesting that the impact of the fire under different vegetation types did not significantly affect the biological response.

## 5. Conclusions

In the investigated Mediterranean area, the comparison of unburnt and burnt sites under the same vegetation type suggested that the effects of fire are greater under P than under H soils, according to the biological responses evaluated by the IBR index. Vegetation type slightly affected the soil biological response before the fire, but its effects increased after the fire. In fact, before the fire, the only soil properties that meaningfully varied between H and P soils were OM content and N concentrations. Instead, after the fire, the differences in the biotic and abiotic properties among the four vegetation types were wider and often significant. Particularly, H soils clearly distinguished from P, HO, and BL, according to the investigated soil properties. In addition, the main abiotic properties that drove the biotic ones were pH, OM, WC, and C/N.

## Figures and Tables

**Table 1:** Characteristics of the soils sampled inside the Vesuvius National Park.

Sites	Sampling Campaign		Coordinates	Plant Cover
	2016 (BF)	2018 (AF)		
H_1	X	X	40°81′31″ 14°43′66″	
H_2 H_3 H_4	X X X	X X X	40°81′81″ 14°43′50″ 40°82′17″ 14°43′57″ 40°82′30″ 14°39′96″	Mosses, lichens, *Centranthus ruber* L., *Helichrysum italicum* R., *Artemisia campestris* L., *Rumex scutatus* L.
H_5	X	X	40°83′07″ 14°25′28″	
P_1	X	X	40°83′10″ 14°25′02″	
P_2 P_3 P_4	X X X	X X X	40° 82′ 41″ 14°39′18″ 40°80′19″ 14°26′13″ 40°79′71″ 14°43′87″	*Pinus pinea* A., (Pine)
P_5	X	X	40°80′19″ 14°43′85″	
BL_1		X	40°81′20″ 14°44′07″	
BL_2 BL_3 BL_4		X X X	40°80′88″ 14°43′92″ 40°82′86″ 14°43′04″ 40°82′36″ 14°43′53″	*Robinia pseudoacacia* L. (*Black locust*)
BL_5		X	40°82′13″ 14°43′62″	
HO_1		X	40°80′72″ 14°43′46″	
HO_2 HO_3		X X	40°80′88″ 14°43′92″ 40°81′03″ 14°40′86″	*Quercus ilex* L. (*Holm oak*)
HO_4		X	40°81′67″ 14°40′86″	
HO_5		X	40°81′99″ 14°39′96″	

**Table 2:** Coefficient of Spearman’s correlation performed between physico-chemical and biological parameters of the soils sampled before the fire.

Pines	C_mic_	C_fung_	BR	HA	DHA	qCO_2_
pH	**0.281**	**−0.194**	**−0.130**	−0.021	−0.130	0.0216
WC	**−0.615**	**0.832**	**−0.874**	**0.930**	**0.837**	**0.685**
OM	**−0.490**	**0.748**	**−0.965**	**0.832**	**0.754**	**0.783**
N	**0.699**	**−0.818**	**0.734**	**−0.860**	**−0.908**	**−0.469**
C/N	**0.399**	**−0.329**	**0.315**	**−0.245**	**−0.373**	**−0.168**
Herbaceous						
pH	−0.151	**0.347**	**−0.440**	**−0.217**	**−0.324**	0.178
WC	**−0.308**	**0.400**	**−0.018**	**0.898**	**0.867**	**0.345**
OM	**0.427**	**−0.427**	0.209	**−0.867**	**0.783**	**−0.255**
N	**−0.006**	−0.100	−0.109	**−0.249**	**−0.301**	**−0.300**
C/N	**0.357**	**−0.255**	**0.264**	**−0.740**	**−0.657**	**0.027**

In bold are the statistically significant correlations.

**Table 3:** Coefficient of Spearman’s correlation performed between physico-chemical and biological parameters of the soils sampled after the fire.

Pines	C_mic_	C_fung_	BR	HA	DHA	qCO_2_
pH	**0.546**	**0.496**	**0.442**	**0.260**	**0.439**	−0.025
WC	−0.003	**−0.359**	**−0.260**	−0.012	**−0.207**	−0.082
OM	0.010	−0.137	0.009	0.012	**0.214**	0.039
N	**0.472**	**0.346**	**0.567**	**0.254**	**0.418**	**0.343**
C/N	**−0.262**	**−0.218**	**−0.343**	−0.051	**−0.260**	**−0.182**
Herbaceous						
pH	**0.243**	**0.316**	−0.108	0.039	**0.353**	−0.104
WC	**0.308**	**0.398**	**0.226**	**0.183**	0.110	0.128
OM	**0.376**	**0.296**	**0.337**	**0.501**	**0.352**	−0.045
N	**0.769**	**0.731**	0.056	**0.803**	**0.571**	**−0.519**
C/N	**0.353**	0.111	**0.600**	**0.486**	**0.689**	−0.040
Holm oak						
pH	0.140	−0.008	0.027	**0.330**	**−0.203**	**−0.392**
WC	**0.283**	**−0.320**	0.147	**0.444**	**0.465**	**−0.202**
OM	0.138	**−0.266**	**0.326**	**0.206**	**0.531**	0.131
N	0.131	**−0.310**	**0.465**	**0.314**	**0.702**	0.076
C/N	0.072	**−0.535**	**0.362**	0.140	**0.365**	0.060
Black locust						
pH	**−0.433**	**−0.346**	**0.051**	**−0.633**	−0.117	0.107
WC	−0.040	0.132	**0.110**	**0.553**	0.088	**0.291**
OM	**0.232**	**−0.186**	**−0.055**	**0.277**	**0.171**	0.090
N	**0.691**	**0.546**	**0.240**	**0.254**	**0.312**	**−0.231**
C/N	**0.377**	**0.219**	0.116	0.093	−0.001	**−0.422**

In bold are the statistically significant correlations.

**Table 4:** Values of physico-chemical and biological parameters of the soils collected before and after the fire under herbaceous and pine specimens.

	Herbaceous (H)		Pines (P)	
	BF	AF	BF	AF
**pH**	7.26	7.37	6.92	7.46
**WC** (% d.w.)	36.3	9.30	46.2	11.7
**OM** (% d.w.)	5.20	3.30	18.7	11.7
**N** (% d.w.)	0.31	0.20	0.61	0.20
**C/N**	11.5	9.6	17.8	16.6
**C_fung_** (mg g^−1^ d.w.)	0.13	0.13	0.02	0.05
**C_mic_** (mg g^−1^ d.w.)	0.80	1.02	1.13	1.60
**BR** (mg CO_2_ g^−1^ d.w.)	0.05	0.05	0.14	0.13
**qCO_2_** (mg C-CO_2_ mg^−1^ C_mic_)	0.10	1.21	0.17	0.03
**HA** (mmol FDA min^−1^ g^−1^ d.w.)	0.16	0.03	0.28	0.20
**DHA** (mmol TPF min^−1^ g^−1^ d.w.)	0.43	5.60	0.50	9.70

## Data Availability

The data presented in this study are available on request from the corresponding author. The data are not publicly available due to the privacy.
